# Physiotherapy as part of primary health care, Italy

**DOI:** 10.2471/BLT.22.288339

**Published:** 2022-09-02

**Authors:** Alessandra Da Ros, Matteo Paci, Elisa Buonandi, Laura Rosiello, Sandra Moretti, Chiara Barchielli

**Affiliations:** a Scuola Superiore Sant’Anna, Istituto di Management, Laboratorio Management e Sanità, Piazza Martiri della Libertà, 33, 56127 Pisa, Italy.; b Dipartimento delle Professioni Tecnico-Sanitarie, Azienda USL Toscana Centro, Florence, Italy.

## Abstract

**Objective:**

To describe the Family and Community Physiotherapist model, which aims to incorporate rehabilitation services within primary health care in Tuscany, Italy.

**Methods:**

The Department of Health Professions of the Central Tuscany local health authority designed the model during 2020–2021. We describe the four phases of the organizational case study implementation of the model, namely: (i) analysis of the political and organizational framework, as well as determination of changing health-care needs; (ii) model co-design and training of multiprofessional health-care workers (local general practitioners, physiatrists and geriatricians); (iii) delivery and surveillance of rehabilitation services; and (iv) evaluation.

**Findings:**

During the initial roll-out of the project in April–December 2021, general practitioners referred 165 patients with a mean age of 83.7 years (standard deviation: 11.1) to the Family and Community Physiotherapist. Interventions were mainly activated for patients with comorbidities (64/165; 38.8%), followed by those with long-term immobilization issues (36/165; 21.8%). The most commonly provided intervention was counselling, contributing to the achievement of objectives for 127 patients (77.0%). A full rehabilitation path was proposed for only 10 patients (6.1%). No additional costs were incurred by the health authority during the implementation of the model.

**Conclusion:**

Our model facilitated the provision of rehabilitative care in the community, preventing the exacerbation of chronic conditions and meeting the population health needs in non-hospital environments. The model overcame the typical lack of integration within health-care services with flexibility, promoting care proximity solutions to cope with health challenges such as an ageing population and the coronavirus disease.

## Résumé

**Objectif:**

Expliquer en quoi consiste le modèle de Kinésithérapeute de famille et de communauté, qui vise à intégrer les services de réadaptation dans les soins primaires en Toscane (Italie).

**Méthodes:**

Le Département des professions de la santé, sous l'égide des autorités sanitaires locales de Toscane centrale, a conçu ce modèle en 2020–2021. Dans le présent document, nous détaillons les quatre phases de l'étude de cas sur l'organisation et la mise en œuvre du modèle, à savoir: (i) l'analyse du cadre politique et organisationnel, ainsi que la définition des besoins changeants en matière de santé; (ii) la conception conjointe du modèle et la formation de professionnels de la santé multidisciplinaires (spécialistes de la réadaptation, gériatres et généralistes locaux); (iii) la prestation et le suivi des services de réadaptation; et enfin, (iv) l'évaluation.

**Résultats:**

Pendant le déploiement initial du projet, entre avril et décembre 2021, les médecins généralistes ont transféré 165 patients dont l'âge moyen s'élevait à 83,7 ans (écart type: 11,1) au kinésithérapeute de famille et de communauté. La plupart des interventions ont été activées pour les patients souffrant de comorbidités (64/165; 38,8%), puis pour ceux présentant des problèmes liés à une immobilisation de longue durée (36/165; 21,8%). Les interventions les plus fréquentes étaient des consultations, qui ont aidé 127 patients (77,0%) à atteindre leurs objectifs. Seuls 10 patients (6,1%) se sont vu proposer un parcours de réadaptation complet. Les autorités sanitaires n'ont engagé aucune dépense supplémentaire durant la mise en œuvre du modèle.

**Conclusion:**

Notre modèle a facilité l'accès aux soins de réadaptation au sein de la communauté, et permis d'éviter l'aggravation d'affections chroniques tout en répondant aux besoins de la population en matière de santé, ailleurs qu'en milieu hospitalier. Il a également surmonté l'absence d'intégration aux services de santé grâce à sa flexibilité, en privilégiant les soins de proximité face à des défis tels que le vieillissement de la population et la maladie à coronavirus.

## Resumen

**Objetivo:**

Describir el modelo de Fisioterapeuta de familia y comunidad, que tiene como objetivo incorporar los servicios de rehabilitación dentro de la atención primaria de salud en la Toscana, Italia.

**Métodos:**

El Departamento de Profesiones Sanitarias de la autoridad sanitaria local de la Toscana central diseñó el modelo durante 2020 y 2021. Se describen las cuatro fases del estudio de caso organizativo sobre la implementación del modelo, a saber: (i) el análisis del marco político y organizativo, así como la determinación de las necesidades cambiantes de atención sanitaria; (ii) el codiseño del modelo y la formación del personal sanitario multiprofesional (médicos generales locales, médicos rehabilitadores y geriatras); (iii) la prestación y la vigilancia de los servicios de rehabilitación; y (iv) la evaluación.

**Resultados:**

Durante el despliegue inicial del proyecto entre abril y diciembre de 2021, los médicos generales remitieron al fisioterapeuta de familia y comunitario a 165 pacientes con una edad media de 83,7 años (desviación estándar: 11,1). Las intervenciones se aplicaron principalmente a los pacientes con comorbilidades (64/165; 38,8 %), seguidos por aquellos con problemas de inmovilización a largo plazo (36/165; 21,8 %). La intervención que más se proporcionó fue el asesoramiento, que contribuyó al logro de los objetivos para 127 pacientes (77,0 %). Se propuso una vía de rehabilitación completa solo para 10 pacientes (6,1 %). La autoridad sanitaria no incurrió en ningún coste adicional durante la implementación del modelo.

**Conclusión:**

Este modelo facilitó la prestación de cuidados de rehabilitación en la comunidad, lo que permitió prevenir el empeoramiento de enfermedades crónicas y satisfacer las necesidades sanitarias de la población en entornos no hospitalarios. El modelo superó la falta de integración que suele haber en los servicios sanitarios con flexibilidad, promoviendo soluciones de proximidad asistencial para abordar dificultades en materia de salud como el envejecimiento de la población y la enfermedad del coronavirus.

## ملخص

**الغرض:**

وصف نموذج أخصائي العلاج الطبيعي للأسرة والمجتمع، والذي يهدف إلى دمج خدمات إعادة التأهيل في الرعاية الصحية الأولية في توسكاني بإيطاليا.

**الطريقة:**

قام قسم المهن الصحية التابع للهيئة الصحية المحلية في توسكاني المركزية بتصميم النموذج خلال الفترة 2020-2021. نحن نقوم بوصف المراحل الأربع لتنفيذ دراسة الحالة التنظيمية للنموذج، وهي: (1) تحليل إطار العمل السياسي والتنظيمي، وكذلك تحديد احتياجات الرعاية الصحية المتغيرة؛ و(2) تصميم نموذج مشترك وتدريب العاملين في مجال الرعاية الصحية ذوي المهن المتعددة (المحللين من الممارسين العموميين، وأخصائيي العلاج الطبيعي، وأطباء الشيخوخة)؛ و(3) تقديم خدمات إعادة التأهيل ومراقبتها؛ و(4) التقييم.

**النتائج:**

خلال الانطلاق الأولي للمشروع في الفترة أبريل/نيسان إلى ديسمبر/كانون أول 2021، قام الممارسون العموميون بإحالة 165 مريضًا بمتوسط عمر 83.7 عامًا (الانحراف المعياري: 11.1) إلى أخصائي العلاج الطبيعي للأسرة والمجتمع. تم تنشيط التدخلات بشكل رئيسي للمرضى المصابين بأمراض مصاحبة (64/165؛(38.8 % ، يليها المرضى الذين يعانون من مشاكل عدم الحركة على المدى الطويل (36/165؛(21.8 % . كان التدخل الأكثر شيوعًا هو تقديم المشورة، مما ساهم في تحقيق الأهداف لعدد 127 مريضًا(77.0 % ) . تم اقتراح مسار كامل لإعادة التأهيل لعدد 10 مرضى فقط(6.1 %) . لم يتم تحميل أية تكاليف إضافية على الهيئة الصحية أثناء تنفيذ النموذج.

**الاستنتاج:**

أدى النموذج الخاص بنا لتوفير رعاية إعادة التأهيل في المجتمع،  حيث منع تفاقم الحالات المزمنة، وقام بتلبية الاحتياجات الصحية للسكان في البيئات خارج المستشفى. تغلب النموذج على النقص المعتاد لتكامل خدمات الرعاية الصحية مع المرونة، وتعزيز حلول القرب من الرعاية، لمواكبة التحديات الصحية مثل السكان الذين يعانون من الشيخوخة، ومرض فيروس كورونا.

## 摘要

**目的:**

描述家庭和社区理疗师模型，该模型旨在将理疗服务纳入意大利托斯卡纳的初级医疗护理之中。

**方法:**

托斯卡纳中央地方卫生局卫生专业部门在 2020-2021 年设计一个模型。我们描述了该模型的组织案例研究实施的四个阶段，即: (I) 分析政治和组织框架，并确定不断变化的医疗护理需要；(ii) 共同设计和培训跨专业医疗护理工作者（当地全科医生、理疗医生和老年医生）的模式；(iii) 提供和监察理疗服务；(iv) 评估。

**结果:**

在 2021 年 4 月至 12 月该项目最初推出期间，全科医生将 165 名平均年龄为 83.7 岁（标准差：11.1）的患者转诊给家庭和社区理疗师。干预措施主要针对有并发症的患者（64/165；38.8%），其次是有长期固定问题的患者（36/165；21.8%）。最常见的干预措施是咨询，帮助 127 名患者 (77.0%) 实现目标。只对 10 名患者 (6.1%) 提出了完整的理疗路径。在实施该模型的过程中，卫生当局没有产生任何额外费用。

**结论:**

该模型促进了社区康复服务的开展，预防了慢性病的加重，满足了非医院环境下人群的健康需求。该模式灵活地克服了医疗护理服务内部缺乏整合的典型问题，促进了就近护理解决方案，以应对人口老龄化和冠状病毒病等健康挑战。

## Резюме

**Цель:**

Описать модель семейного и общинного физиотерапевта, целью которой является включение реабилитационных услуг в первичную медико-санитарную помощь в Тоскане, Италия.

**Методы:**

В 2020–2021 гг. Департамент медицинских профессий местного органа здравоохранения Центральной Тосканы разработал такую модель. Авторы описывают четыре этапа организационной реализации тематического исследования данной модели, а именно: (i) анализ политической и организационной структуры, а также определение меняющихся медико-санитарных потребностей; (ii) модель совместной разработки и обучения многопрофильных медицинских работников (местных врачей общей практики, физиотерапевтов и гериатров); (iii) предоставление и надзор за реабилитационными услугами; (iv) оценка.

**Результаты:**

Во время первоначального осуществления проекта в апреле-декабре 2021 года врачи общей практики направили 165 пациентов со средним возрастом 83,7 года (стандартное отклонение: 11,1) к семейному и общинному физиотерапевту. Меры были в основном осуществлены для пациентов с сопутствующими заболеваниями (64/165; 38,8%), за которыми следовали пациенты с проблемами длительной иммобилизации (36/165; 21,8%). Наиболее частой мерой было консультирование, которое способствовало достижению целей у 127 пациентов (77,0%). Полный путь реабилитации был предложен только 10 пациентам (6,1%). Во время внедрения модели орган здравоохранения не понес никаких дополнительных затрат.

**Вывод:**

Данная модель помогла оказать реабилитационную помощь в местных общинах, предотвратить обострения хронических состояний и удовлетворить потребности населения в охране здоровья в амбулаторных условиях. Эта модель позволила гибко преодолеть типичное отсутствие интеграции в рамках медицинских услуг, содействуя решению вопросов, связанных с обеспечением доступности медицинской помощи, и разрешать такие сложные проблемы в области здравоохранения, как старение населения и коронавирусная болезнь.

## Introduction

Rehabilitation is one of the five pillars of universal health coverage, together with health promotion, the prevention and treatment of disease, and palliative care.^1^ Function, one of the most important indicators of health after mortality and morbidity, is optimized by rehabilitation.^2^ This pillar is usually confined to tertiary (i.e. specialized) health-care facilities because of its intrinsic multidimensional and complex nature.^3^ However, there exist several provisional models to integrate rehabilitation within primary health-care services.^4^

As defined in the proceedings of the Alma Ata Conference in 1978,^5^ primary health-care services refer to the first contact between patients and health-care professionals.^1^ The pivotal role of primary health care was also confirmed by the recent Declaration of Astana in 2018, in which states and governments strongly envision that primary health care “will be implemented in accordance with national legislation, contexts and priorities…to avoid fragmentation and ensure a functional referral system between primary and other levels of care.”^6^

Since it has been estimated that at least one third of the global population will need rehabilitation at some point over the course of their illness or injury, the strengthening of rehabilitation to include the early identification of health needs and referral reduces the impact of disabling effects and optimizes function.^7^ Integrated rehabilitation services and innovative rehabilitation provision delivery models will benefit all levels of care, in both urban and remote areas. The World Health Organization Rehabilitation 2030 initiative already advocates for strategies to reinforce rehabilitation to support the changing health needs of the ageing global population, that is, an increased incidence of new and chronic impairments.^8^ Further, rehabilitation contributes to the achievement of the sustainable development goal 3 – ensure healthy lives and promote well-being for all at all ages.^9^ The effects of the coronavirus disease 2019 (COVID-19) pandemic, with the accompanying difficulties experienced in accessing services and the long-term health consequences, make this goal particularly relevant.^10^ Health-care professionals therefore propose the strengthening of rehabilitation services at the primary health-care level.^7^

Models of rehabilitation services describe a set of organizational methods to achieve a common health-care objective; there is no absolute best model, but rather a best model to address the needs of a particular population group. This variability allows flexibility within an organization and the best possible use of human resources in each specific context. The key elements of existing rehabilitation service models include: (i) coordination within a network of health-care professionals; (ii) the possibility of patient empowerment and independence; and (iii) community involvement, creating informal networks.^4^

The main barrier to the implementation of rehabilitation models in primary health care is the lack of service integration, which requires collaboration and communication between professionals. To promote organizational change and scale up innovations across different national and international settings, rehabilitation models must consider the health needs of future populations while also sharing experiences, knowledge, resources and professional competencies. We therefore designed the novel rehabilitation service model, the Family and Community Physiotherapist initiative, introduced in Tuscany, Italy in 2021. We report on the model implementation phases and describe the outcomes achieved towards the aim of integrating rehabilitation within primary health-care services.

## Methods

We followed the theory of an organizational case study^11^^,^^12^ in developing the four phases of implementation of our Family and Community Physiotherapist model (Box 1), namely: analysis of political framework and health needs; model design and health-care professional training; service delivery; and evaluation. Relying on Mintzberg’s organizational framework,^13^ our model can be classified under the umbrella of adhocracy configuration: it drives innovation through expert teamwork integration and specialized competencies within complex organizations.

Box 1 **Phases of the Family and Community Physiotherapist model implementation, Tuscany, Italy, 2021**

- Phase 1: preliminary determination of emerging health-care needs, with analysis of the relevant political and organizational frameworks
- Phase 2: innovative model design and advanced training of network of multiprofessional health-care workers
- Phase 3: autonomous delivery and surveillance of service
- Phase 4: data evaluation, experience sharing and future scale-up planning

### Political framework analysis

The Italian government presented its six-mission Recovery and Resilience Plan to the European Commission in 2021, articulating necessary reforms and interventions to respond to the unprecedented COVID-19 pandemic.^14^ The sixth mission dealt with health-service policies, including challenges in improving access to health-care services within the community and in overcoming significant disparities across the 20 Italian regions.

Since 2018, the social and health policies of the region of Tuscany have been focused on the integration of care provision to chronically ill patients within primary health-care services, as opposed to hospital care. The first local pilot experience that brought professionals closer to patients and the whole community was the Family and Community Nurse model, inspired by the earlier Chronic Care Model,^15^ based on: (i) proximity of the health-care professional to a reference population; (ii) transversality of competencies and dedicated specialist competencies to be activated when needed (e.g. home ventilation, pressure ulcer treatment, etc.); (iii) the provision of information on health-care availability; and (iv) proactivity of the health-care worker through an analysis of patients’ latent needs.^16^

As part of the Department of Health Professions of the Central Tuscany local health authority (*Unità Sanitaria Locale Toscana Centro*; that serves four homogenous functional territorial units totalling 1.7 million people over an area of 5000 km^2^), we developed the Family and Community Physiotherapist model in 2020. This model was based on the previous Family and Community Nurse model, and was a continuation of an experimental model that had been approved at the regional level in mid-2019.^17^ In the first phase of the model, we held online brainstorming meetings in December 2020 to explore post-COVID-19 rehabilitation needs with the Head of the Department of Health Professions (a radiology technician) and the Head of the Organizational Innovation Office (a nurse) within the Nursing and Midwifery Department (in which the Family and Community Nurse model had been implemented).^16^^,^^18^

### Model design

The second phase dealt with the organizational model co-design and the definition of the appropriate competences. Following our initial online brainstorming meetings, we discussed emerging features of the model in face-to-face focus groups with executives of the Department of Health Professions (physiotherapists), physiotherapist managers and physiotherapists with clinical roles in January 2021. We then shared a digital version of the manuscript draft, asking all participants for comment. During subsequent meetings held in the spring of 2021, we incorporated feedback to produce an assessment checklist for patients for distribution to local general practitioners, physiatrists (the medical doctor responsible for defining and coordinating the patient’s rehabilitation project), and geriatricians. Our checklist classifies the health needs of the target population as: (i) significant reduction in functional autonomy; (ii) increased care burden or need for caregiver training; (iii) history of falls/risk of falling; and/or (iv) need for home environment assessment.

As part of the national programme of continuous training, we invited all physiotherapists employed by the local health authority in the area of domiciliary care to attend training in the Family and Community Physiotherapist model. The Department of Health Professions funded and delivered training in specific skills, such as: analysis of the health needs of individuals, families and communities; promotion, planning and supervision of rehabilitation treatment through prevention activities and educational interventions; and activation of the most appropriate team assistance or social network. We invited those physiotherapists who had completed the relevant training to volunteer for participation in the service delivery. 

### Service delivery

Unlike the Family and Community Nurse model, in which each nurse provided care services to a fixed register of around 2500 members of the population, the Physiotherapist model is an on-call service requested by general practitioners. During the implementation phase of the model, on the identification of patient needs in one of the four areas mentioned above, general practitioners would request an intervention from the Family and Community Physiotherapist.^19^ The model envisages a maximum of three in-person interventions per patient, as well as the possibility of remote access (e.g. by telephone or video call). We initially allocated 12 hours per working week of each full-time participating physiotherapist to the rehabilitation service. 

Typical interventions include the provision of counselling to families and caregivers on mobility aids and equipment, strategies to reduce the risk of falls, and approaches to support and maintain patient functionality. However, if the Family and Community Physiotherapist identifies the need for more extensive rehabilitation interventions or the intervention of other team professionals, they can ask for access to the most appropriate network of services through specific pathways.

### Evaluation

Finally, in the last phase of the project we used quantitative data extracted from a computerized ad hoc data flow administered by the executives and managers of the local health authority to monitor the model implementation and the health outcomes of the served population. Data obtained from patients’ clinical records were recorded by health-care professionals and monitored by physiotherapist managers.

## Results

During the initial roll-out of the project in April–December 2021, general practitioners referred 165 patients (Table 1) with a mean age of 83.7 years (standard deviation, SD: 11.1) to the family and community physiotherapist. Interventions were activated within a mean of 15 days (SD: 22.7) from the initial referral, and mostly for patients with comorbidities (64/165; 38.8%) followed by those with long-term immobilization issues (36/165; 21.8%).

**Table 1:** Characteristics of patients included in the initial roll-out of the Family and Community Physiotherapist model, Tuscany, Italy, April–December 2021

Characteristic	No. (%) (*n* = 165)
**Sex**	
Male	65 (39.4)
Female	100 (60.6)
**Principal medical diagnosis**	
Multi-pathological	64 (38.8)
Long-term immobilization consequences	36 (21.8)
Neurological	25 (15.2)
Frail patient	18 (10.9)
Musculoskeletal	13 (7.9)
Oncological	1 (0.6)
Other	8 (4.8)

The services required and health outcomes of these patients are described in Table 2. Since its implementation, the model was activated primarily to address reduction in a patient’s functionality. With a mean of around three interventions (2.6; SD: 1.3) per patient, the most provided intervention was counselling, contributing to the appropriate achievement of the objectives set for 127 patients (77.0%) with no adverse events reported (Table 2). The introduction of remote sessions (1.1; SD: 0.8 per patient compared with 1.7; SD: 0.9 of in-person sessions per patient) to facilitate the connection between health-care professional and patient, enhancing the proximity of care, was particularly appreciated by the population. 

**Table 2:** Health-care needs of, and services accessed by, patients included in the initial roll-out of the Family and Community Physiotherapist model, Tuscany, Italy, April–December 2021

Intervention	No. (%) (*n* = 165)
**Achieved goals, appropriateness^a^**	127 (77.0)
**Consultancy setting**	
Clinic	10 (6.1)
Patient’s home	155 (93.9)
**Consultancy activation area**	
Significant reduction in functional autonomy	98 (59.4)
Increased care burden or need for caregiver training	36 (21.8)
Home environment assessment	18 (10.9)
History of falls/risk of falling	13 (7.9)
**Provided intervention**	
Counselling	98 (59.4)
Review of mobility aids and equipment	18 (10.9)
Full rehabilitation path	10 (6.1)
Caregiver training	10 (6.1)
Falling prevention training	5 (3.0)
Other	24 (14.5)

^a^ As determined by the general practitioners who referred patients to the rehabilitation service.

The prevalence of home interventions supports the need for a flexible approach in health care, such as that proposed by the model, in which rehabilitation treatment can be provided within a primary health-care network. A full rehabilitation path with specialist activation was proposed for only 10 patients (6.1%), supporting the hypothesis of the benefit of a consultancy rehabilitation health-care worker that acts as a link to specialist care.

The local authority incurred no additional costs during the implementation of the model.

## Discussion

A functioning health-care system should provide a flexible vision of cooperation (i.e. between hospitals and community health workers) to provide effective, resilient and egalitarian responses to future challenges or health demands expressed by the population, especially by elder or fragile patients or patients with multiple chronic conditions. The Family and Community Physiotherapist model roll-out in Tuscany enabled the integration of rehabilitation within primary health care, facilitating the provision of rehabilitative care in the community, preventing the exacerbation of chronic conditions, and meeting the health needs of the population in non-hospital environments as incentivized during the COVID-19 pandemic.^20^^–^^22^ The close collaboration of physiotherapists with general practitioners, physiatrists and geriatricians enabled the interventions to be prompt, shared, accurate, appropriate and, above all, safe.^23^^,^^24^ The disease prevention aim of the model allows the delivery of chronic disease management outside of the hospital environment, before the pathology arises or worsens.^21^^,^^22^

As demonstrated, the Family and Community Physiotherapist model does not require any financial investment, but has a potentially high economic impact in terms of preserved or regained patient functionality. The proposed model is consistent with emerging evidence on the value-for-money rehabilitation services delivered in patients’ homes in most European countries.^25^ By targeting high-risk patients in primary health care, the model also helps to avoid the cost of hospitalizations or re-admissions by mitigating the risk of preventable complications.^26^^,^^27^

The sharing of innovative models and their frameworks across the field can be useful to promote organizational change and scale up innovations to achieve global goals.^8^^,^^9^ Similarly, our model allows widespread scalability: it describes a best organizational practice that can be tailored to different contexts and professionals (e.g. dietician, X-ray technician, etc.). By taking advantage of available finance (i.e. Recovery and Resilience Plan), other Italian regions could establish the required human and technical resources to benefit from implementation of this model. The proposed model could also be easily exported to various other socioeconomic (e.g. to low-, middle- and high-income countries) and environmental (e.g. urban centres, mountainous regions and rural areas) contexts, or adapted for specific circumstances (e.g. the COVID-19 pandemic or other emergencies) so that more patients can benefit.^7^ The model does not need to be supported by complex or major tertiary-level health-care structures (e.g. large hospitals), but should work alongside general practitioners at the community level. Scientific associations and professional orders could adopt the strategic role of sponsoring and supporting the presented model and its underlying values.

A further incentive to implementation of this model has been highlighted by the pandemic, and relies on technological and digital advancement.^28^ Remote services supported by telehealth (i.e. teleconsultations, telerehabilitation) emerge as tools that the family and community health-care workers could use extensively to enhance their presence and proximity to patients. Primary health-care teleconsultation is effective and secure, while reducing the time burden for patients, families and professionals.^29^^,^^30^

In the complex scenario of the Italian health-care system, the lesson learnt is that innovative solutions can and should be implemented to meet the global pace of change. Prompt field research and experience sharing play a pivotal role in producing evidence to inform policy on how to respond to increasing rehabilitative needs.^31^ International policy and comparable data flows enabling the quantification of performance evaluation indicators are fundamental to determine future human resources needs and standards to create a network of skilled professionals.^32^

Despite its obvious advantages, there exists a main barrier to this type of health-care model: a lack of rehabilitative health services integration vision in primary health care as a result of acute health-care service historical centredness.^21^ This mindset creates a vicious circle in which health-care services deal with consequences and not causes. Although hospitals are commonly recognized hubs for health issues, and health-care professionals feel comfortable in this setting, health and functionality are something that can be considered as beginning within the patient’s home.^33^ For this reason, as with other prevention strategies, the benefits are not immediately visible; however, introducing such models simplifies and accelerates the patient pathway towards the highest grade of health and well-being achievable from primary health care.

Our model would benefit from the collection of feedback from patients and caregivers on the perceived experience of care,^34^ as well as from health-care professionals on their levels of satisfaction with the model provided.^23^^,^^35^ These future evaluations have the potential to meet health-care challenges further, while ensuring the quality of care and people-centredness. Policy-makers could then promote a broad communication campaign, raising public awareness among citizens of the provision of rehabilitation services within primary health care.

Our results demonstrate that the Family and Community Physiotherapist model is of great benefit to the population of the Central Tuscany local health authority, and has the potential to be scaled up or tailored to other environments and health needs. The organizational innovation provides rehabilitation health services at the population level, together with acute and post-acute care. The innovative rehabilitation model overcomes the typical lack of integration within health-care services with flexibility, promoting care proximity solutions to cope with expected (i.e. demographic ageing) and unexpected (i.e. COVID-19) health challenges.
